# Web-Based Medical Examinations During the COVID-19 Era: Reconsidering Learning as the Main Goal of Examination

**DOI:** 10.2196/25355

**Published:** 2021-08-09

**Authors:** Amirreza Manteghinejad

**Affiliations:** 1 Cancer Prevention Research Center Omid Hospital Isfahan University of Medical Sciences Isfahan Iran; 2 Student Research Committee School of Medicine Isfahan University of Medical Sciences Isfahan Iran

**Keywords:** COVID-19, online exam, e-learning, medical education, medical student, online learning, online platform, cheating, web-based examination

## Abstract

Like other aspects of the health care system, medical education has been greatly affected by the COVID-19 pandemic. To follow the requirements of lockdown and virtual education, the performance of students has been evaluated via web-based examinations. Although this shift to web-based examinations was inevitable, other mental, educational, and technical aspects should be considered to ensure the efficiency and accuracy of this type of evaluation in this era. The easiest way to address the new challenges is to administer traditional questions via a web-based platform. However, more factors should be accounted for when designing web-based examinations during the COVID-19 era. This article presents an approach in which the opportunity created by the pandemic is used as a basis to reconsider learning as the main goal of web-based examinations. The approach suggests using open-book examinations, using questions that require high cognitive domains, using real clinical scenarios, developing more comprehensive examination blueprints, using advanced platforms for web-based questions, and providing feedback in web-based examinations to ensure that the examinees have acquired the minimum competency levels defined in the course objectives.

## Background

Currently, we are living in the COVID-19 era [1], during which almost every aspect of life, from communications to people’s lifestyles, has changed [2,3]. Originating in China, the SARS-CoV-2 virus spread worldwide quickly, affected billions of people, and led to several infection control policies, such as wearing masks, social distancing, and lockdowns [4-6]. Iran is one of the countries that was greatly affected by this pandemic. The first cases of COVID-19 were reported on February 19, 2020, and to date, approximately 2.8 million confirmed cases and 78,000 deaths have been reported in Iran. Medical education, including undergraduate and postgraduate education, was not immune to the effects of this virus [7]. A sudden shift to e-learning and web-based courses was a direct result of COVID-19 [8]. For undergraduate students, almost all courses are presented virtually, and for postgraduate students, a blended learning approach is used to reduce the exposure of students, interns, and residents to the disease. Traditional written examinations also underwent a transformation in response to this pandemic, considering the high risk of infection in indoor testing sites [9,10]. The easiest way to face the new challenges was to administer traditional questions via a web-based platform. However, more factors should be accounted for in designing web-based examinations during the COVID-19 era. This article aims to address some of the major aspects that should be considered in this regard and to suggest a valid and reliable method for organizing medical students’ examinations in the COVID-19 era.

## COVID-19 and Web-Based Examinations

Resorting to web-based examinations during the COVID-19 pandemic was inevitable. It should be noted that COVID-19 itself affects medical students, medical educators, and medical universities more than lockdowns and social distancing. Other aspects of COVID-19 that must be taken into account during the pandemic can be categorized into mental aspects, educational aspects, and technical aspects.

### Mental Aspects

Stress and burnout are among the factors that may affect medical students during this pandemic [11]. Studies conducted before the COVID-19 pandemic showed that stress levels were high among medical students [12]. The COVID-19 pandemic has now worsened the stress levels of medical students and workers throughout the entire health care community [13,14]. In our experience in Medical University of Isfahan (MUI) teaching hospitals, some of the main causes of stress during this pandemic were the shortage of personal protective equipment in the early weeks; the poor state of intern and resident on-call rooms, which increased the risk of infection; and the fear of infecting family members.

Regarding burnout, previous studies have shown a higher prevalence of burnout in medical students than in the general population [15]. Currently, during the COVID-19 pandemic, burnout syndrome has become more prevalent in health care providers and medical students [14,16].

### Educational Aspects

The difference in the types of education that medical students, interns, and residents receive also increases the need to change the way examinations are conducted. The sudden shift to e-learning has created challenges in medical education [17]. Challenges include decreased numbers of educational opportunities, decreased numbers of patients (except for patients with COVID-19), increased numbers of shifts, and in some cases, the requirement to work in COVID-19 wards. As a result of the second and third waves of this pandemic in Isfahan, the primary teaching hospital of MUI became a COVID-19 center, and all operation rooms or outpatient clinics were closed, thus decreasing educational opportunities for interns and residents.

### Technical Aspects

Web-based examinations also have unique problems. Studies show that web-based testing environments negatively affect student performance on examinations because of differences in student comfort and technical problems, such as internet connection disruptions and server failure [18]. This problem occurred in our first experiences of web-based examinations at MUI. Server error messages appeared during the examinations due to the lack of server resources and negatively affected the students’ performance by causing them stress or wasting examination time.

The ability to control the environment of unproctored web-based examinations is also questionable [19]; for instance, cheating is a commonly reported challenge in web-based assessments [20]. Kennedy et al [21] reported that 57% of students and 64% of faculty members believed that it is easier to cheat on web-based examinations than on traditional examinations. In another study, Jensen and Thomas [22] showed that approximately 22% of participants in web-based examinations used search engines to find the correct answer. This was also the case in MUI examinations. Our first web-based examination results showed a significant increase in examination grades. Further investigations revealed that using messaging platforms such as WhatsApp to share the answers is one of the most common ways of cheating among students. Some other common ways of web-based cheating are using electronic books and typed handouts and searching for keywords.

In response to all these emerging problems, most solutions are limited to technical aspects and preventing students from cheating. Some educators reduced the examination time or used more difficult questions to address this problem. Using webcams and screen sharing to control students is another solution. This method, however, has certain limitations, such as the limits concerning the number of examination participants and the need for high-speed internet connections for all students [23]. Meanwhile, technology can be employed to prevent cheating. For example, PageFocus is a JavaScript code that detects when participants abandon test pages by switching to another window or browser tab [24]. However, the inclusive use of smartphones and messaging applications undermines this method. Sharing answers on social media platforms can thwart the strategies employed by educators, such as decreasing the duration of examinations. Generally, using a second device can neutralize strategies such as screen sharing and PageFocus.

## Learning as the Main Goal of Examinations

Although all these problems related to web-based examinations in the COVID-19 era seem concerning at first look, this leads us to take a step back and reconsider the aims of an examination. One of the main goals of an examination is to improve learning; however, learning through examination can only occur when the examination involves more than participating in the examination, answering the questions, and waiting for the results.

The COVID-19 era and web-based examinations are providing an excellent opportunity to consider learning as the main goal of examination. By accepting the existing limitations in educating and assessing students and preventing cheating in this era, some measures can be taken to transform the examination sessions into learning sessions to ensure that students have achieved the minimum competency levels required for passing their courses. For this transformation, the following approach is suggested.

## Suggestions

### Use Open-Book Examinations

The reason for suggesting open-book examinations is that it may encourage students to use their books or search for the answers on evidence-based medicine databases. Moreover, a systematic review showed decreased anxiety levels among students in open-book examinations, which makes them more favorable, especially in this era [25]. Evidence-based medicine is a result of the internet revolution, and it emerged because of the rapid expansion of knowledge in the age of information; its primary goal is to educate clinicians on how to use published articles for optimizing clinical care [26]. To use evidence-based medicine, physicians must possess the ability to search for and find correct information on the internet and in medical databases [26]; therefore, open-book examinations are an excellent way to familiarize medical students with evidence-based medicine [27].

### Use Questions That Require High Cognitive Domains

To encourage students to use their books and other evidence-based medicine sources during examinations, it is essential to revise the examination questions.

According to Bloom’s theory, cognition has multiple domains [28]. Open-book examinations enable examiners to ask questions that require higher cognitive domains [29], because the students cannot find the test answers easily in references or on the internet with a simple keyword search. Using questions that require high cognitive domains forces the students to read the related contents thoroughly and ensures that students answering the questions have read the selected contents at least once and have understood them.

Using clinical scenarios is a good way to develop questions for open-book examinations because these scenarios require high cognitive domain levels. Open-book examinations are, in fact, similar to clinical practice in certain aspects. In real practice, general practitioners must have the ability to use evidence-based medicine in their decision-making; therefore, they should know how to search, where to search, and what to search for. Using real clinical scenarios in web-based examinations is an excellent method to train medical students in using evidence-based medicine by searching in evidence-based medicine resources [30].

### Create More Comprehensive Examination Blueprints

We use examinations to ensure that medical students have a minimum competency in the subject of the examination. To achieve this goal, especially when the education system is less than perfect, it is necessary to develop more comprehensive examination blueprints. By using a comprehensive blueprint in coordination with open-book examinations, the instructor ensures that all the students recall, review, read, and understand the course topics and subtopics. A common guideline for cognitive domains of questions is 50-40-10, which means that 50% of questions require the knowledge domain, 40% require the application domain, and 10% require problem-solving skills [31,32]. However, as mentioned above, questions that require high cognitive skills such as problem-solving must be given more weight in these circumstances. The type of examination (formative or summative) and examinee (student, intern, or resident) can also change these percentages to ensure that an examinee who passes the examination has a minimum competency according to the course objectives [33].

### Use Advanced Platforms for Web-Based Examinations

Using advanced platforms to administer web-based examinations makes it difficult for students to cheat and encourages them to refer to their books to find the answers [29]. Showing only one question at a time on screen, sorting the questions and choices randomly for multiple-choice questions, and prohibiting students from revisiting a question can help the instructors encourage students to use references instead of cheating [34-36]. Reducing stress is an additional goal of using advanced platforms. Internet connection disruptions and server failure are major causes of stress during web-based examinations. The web-based examination platform must be capable of handling these disruptions; it should save the students’ previous answers and their remaining examination time to curb the negative effects of disruptions on the performance of the examinees.

### Provide Feedback

Learning in an examination session occurs when the examination requires students to do more than merely participate, answer questions, and wait for their grades. Providing feedback is an essential component to turn an examination session into a learning session. The question-response-feedback approach is one of the easiest ways to create a learning session in web-based examinations [37]. This type of feedback is divided into three categories: indication of a correct or incorrect response, statement of the correct response, and elaborative corrective feedback that includes an explanation of the question and responses. Moreover, the feedback could be provided after each question (immediate) or at the end of the test (delayed). Previous studies show that providing feedback with examination question rationales is a better approach than simply providing the correct answer or simply indicating whether the student was correct or not [37-39]. Moreover, a study shows that delayed feedback in examinations is more beneficial than immediate feedback [37]. In conclusion, using delayed elaborative feedback in web-based examinations is suggested for such examinations in this era.

## Conclusion

There is no doubt that medical education, especially in the clinical setting, is being affected by the COVID-19 pandemic. Therefore, there is a need for new education and examination policies to adapt to this situation. Learning is one of the goals of examination that both instructors and students often neglect. The COVID-19 pandemic era is an excellent opportunity to consider learning as the main goal of exams and use methods to transform an exam session into a learning session and ensure that the students who pass the exam have a minimum competency.

## Abbreviations

MUI: Medical University of Isfahan
